# Fingolimod alters the transcriptome profile of circulating CD4+ cells in multiple sclerosis

**DOI:** 10.1038/srep42087

**Published:** 2017-02-03

**Authors:** Jörg Friess, Michael Hecker, Luisa Roch, Dirk Koczan, Brit Fitzner, Ines Charlotte Angerer, Ina Schröder, Kristin Flechtner, Hans-Jürgen Thiesen, Alexander Winkelmann, Uwe Klaus Zettl

**Affiliations:** 1 University of Rostock, Department of Neurology, Division of Neuroimmunology, Gehlsheimer Str. 20, 18147 Rostock, Germany; 2 Steinbeis Transfer Centre for Proteome Analysis, Schillingallee 70, 18057 Rostock, Germany; 3 University of Rostock, Institute of Immunology, Schillingallee 70, 18057 Rostock, Germany

## Abstract

Multiple sclerosis is a demyelinating disease affecting the central nervous system. T cells are known to contribute to this immune-mediated condition. Fingolimod modulates sphingosine-1-phosphate receptors, thereby preventing the egress of lymphocytes, especially CCR7-expressing CD8+ and CD4+ T cells, from lymphoid tissues. Using Affymetrix Human Transcriptome Arrays (HTA 2.0), we performed a transcriptome profiling analysis of CD4+ cells obtained from the peripheral blood of patients with highly active relapsing-remitting multiple sclerosis. The samples were drawn before the first administration of fingolimod as well as 24 hours and 3 months after the start of therapy. Three months after treatment initiation, 890 genes were found to be differentially expressed with fold-change >2.0 and t-test p-value < 0.001, among them several microRNA precursors. A subset of 272 genes were expressed at lower levels, including CCR7 as expected, while 618 genes showed an increase in expression, e.g., CCR2, CX3CR1, CD39, CD58 as well as LYN, PAK1 and TLR2. To conclude, we studied the gene expression of CD4+ cells to evaluate the effects of fingolimod treatment, and we identified 890 genes to be altered in expression after continuous drug administration. T helper cells circulating in the blood during fingolimod therapy present a distinct gene expression signature.

Multiple sclerosis (MS) is an inflammatory demyelinating disease of the central nervous system (CNS) affecting more than 2.3 million people worldwide. It is a common cause of chronic neurological disability in young adults^1^,^2^. Disease onset is typically between 20 and 40 years of age, with a prevalence 3 times higher for women than for men. The majority of patients (~85%) have the relapsing-remitting form of MS (RRMS), which is characterised by neurological flares (relapses) followed by periods of stability (remission)^3^,^4^.

Genetic and environmental factors are known to contribute to the development of MS^5^,^6^. The pathogenesis of MS includes inflammatory and neurodegenerative mechanisms presumably driven by the migration of autoreactive lymphocytes across the blood-brain barrier (BBB)^7^,^8^,^9^. A dysregulated adaptive immune response by T cells is thought to play a role in MS, leading to demyelination and axonal injury within the CNS. In particular, CD4+ T helper 1 cells (Th1) and CD4+ T helper 17 cells (Th17), which differentiate from naive CD4+ T cells in the presence of the cytokines IL-6, IL-23 and TGF-beta^10^,^11^, were shown to promote neuronal damage and BBB disruption^12^,^13^. Recent studies suggest that microRNA (miRNA) are implicated in T helper cell differentiation^14^. MicroRNA are small non-coding RNA regulating gene expression by binding to mRNA targets, which results in translational inhibition and/or mRNA degradation^15^. There is also growing evidence that miRNA are involved in the pathogenesis of autoimmune diseases, and some miRNA are discussed as biomarkers for MS^16^,^17^.

Several disease-modifying therapies with proven clinical benefits are available for the treatment of RRMS. They allow to reduce the rate and severity of relapses and the number of new brain lesions seen in magnetic resonance imaging (MRI)^18^,^19^. The first approved oral medication for highly active RRMS was fingolimod, a sphingosine-1-phosphate (S1P) receptor modulator^20^,^21^,^22^. Fingolimod has been demonstrated to reduce clinical and MRI disease activity in patients with RRMS, but adverse effects, including a temporary decrease in the heart rate after initial administration, have been reported as well. S1P is a bioactive metabolite formed from sphingosine. It functions as a major regulator of immune cell trafficking^23^. As a structural analogue of natural sphingosine, fingolimod can be phosphorylated to produce fingolimod-phosphate, which binds to S1P receptors expressed on lymphocytes. Whereas S1P binding results in internalisation and recycling of the S1PR1 receptor (S1P1), phosphorylated fingolimod causes prolonged internalisation and degradation, thus acting as a functional antagonist^24^,^25^. Therefore, fingolimod affects the competing chemotactic signalling in secondary lymphoid organs of egress-promoting S1P receptors and homing receptors such as CCR7. In the absence of S1P1, CCR7+ lymphocytes are unable to override the retention signals in lymphoid tissues^23^.

As a consequence, patients treated with fingolimod show a reduction in peripheral lymphocyte counts^26^. After 12 months of therapy, CD19+ B cells are reduced from ~6% to <2% and CD4+ T cells are reduced from ~32% to <6% within the total lymphocyte population^27^. Regarding CD4+ T cells, the absolute counts of naive T cells, central memory T cells (TCM), effector memory T cells (TEM) and regulatory T cells (Treg) were all found to be decreased in peripheral blood during therapy^28^. However, the effects vary considerably for the different cell subpopulations. While the egress of CCR7+ naive T cells and CCR7+ TCM from lymph nodes is strongly inhibited by fingolimod, CCR7- TEM are generally spared^29^,^30^. Moreover, CD4+ T cell subsets producing pro-inflammatory cytokines (IFN-gamma and IL-17) are significantly reduced in response to fingolimod treatment, while the frequency of circulating Treg is increased^31^,^32^. By this means, fingolimod is thought to prevent the infiltration of the CNS by autoaggressive T cells.

The aim of the present study was the evaluation of differential gene expression in response to fingolimod in CD4+ cells obtained from blood samples of RRMS patients in order to achieve a better understanding of MS and the drug’s molecular mechanisms of action. For this purpose, a longitudinal gene expression profiling was performed in the course of fingolimod therapy. Using high-resolution Affymetrix HTA 2.0 microarrays, it was not only possible to quantify all human protein-coding transcripts at the exon level, but also to measure the levels of non-coding transcripts, including precursors of mature miRNA.

## Methods

### Study Population

Ten patients of Western European descent, aged between 26 and 46 years, were asked to participate in this study at the Department of Neurology, University of Rostock. Among them, seven were females and three were males. The patients were diagnosed with highly active RRMS according to the revised McDonald criteria from 2010^33^. Routine medical care was provided to all patients. They were treated and monitored according to the European Medicines Agency labels, following the consensus treatment guidelines and recommendations of the German Society of Neurology. Prior to the study, three of the patients received glatiramer acetate (GA), while seven received interferon-beta (IFN-beta). All patients showed relapses despite immunomodulatory treatment. Therefore, they switched to fingolimod treatment at standard dose of 0.5 mg once daily. Most patients (n = 8) were prescribed fingolimod within one month or less after discontinuing their previous medication. At the start of fingolimod therapy, the patients had a median disease duration of 7 years (range, 1 to 20). On the Expanded Disability Status Scale (EDSS), a measure of functional disability in MS^34^, they reached scores between 1.5 and 5.5 (Table 1).

As part of our research on MS, this study was approved by the University of Rostock’s ethics committee and carried out according to the Declaration of Helsinki. All patients gave written informed consent to participate in this study before the first blood sampling.

### CD4+ Cell Isolation and RNA Extraction

Blood samples were obtained for the longitudinal analysis of the patients’ CD4+ cell transcriptome. A total of 20 ml peripheral venous blood was collected from each of the 10 RRMS patients into EDTA (ethylenediaminetetraacetic acid) blood tubes at three different time points: before the first application of fingolimod (baseline), after 24 hours (before the second application) as well as after 3 months of treatment.

The blood samples were magnetically labelled with Whole Blood CD4 MicroBeads (Miltenyi Biotec, Germany) in order to isolate cells expressing the surface molecule CD4, which is primarily found on T helper cells but also on a subset of monocytes. The CD4+ cells were magnetically separated (positive selection) according to the manufacturer’s instructions using the autoMACS separator (Miltenyi Biotec). The unlabelled fraction was rejected. After addition of lysis buffer, the samples were stored until further use at −20 °C.

Total RNA was extracted using the mirVana isolation kit (Ambion, TX, USA). RNA concentrations were measured using a NanoDrop 1000 spectrophotometer (Thermo Fisher Scientific, DE, USA). RNA integrity numbers (RIN) as estimates of the quality of RNA^35^ were determined with an Agilent 2100 Bioanalyzer using RNA 6000 Pico LabChips (Agilent Technologies, CA, USA).

### Microarray Hybridisation and Data Preprocessing

The cellular RNA was analysed with Human Transcriptome Arrays (HTA) 2.0, the latest generation of Affymetrix microarrays, which contain more than six million distinct oligonucleotide probes (25 bases in length) covering >67,500 coding and non-coding transcripts^36^. Approximately 70 percent of the probes on these high-resolution GeneChip microarrays match to the exons of protein-coding transcripts and the remaining 30 percent match to exon-exon splice junctions and non-coding transcripts according to the current gene annotation. The probes can be summarised into gene level, exon level and splice junction probe sets. HTA 2.0 microarrays are designed with 10 probes for each unique exon fragment (PSR) and 4 probes per exon-exon splice junction (JUC). This high coverage provides deep insights into basically all human transcripts^36^.

From each of the 30 samples, cRNA was prepared from 70 ng total RNA according to the Affymetrix Whole Transcript (WT) protocol. The cRNA was used to generate single-stranded DNA, which was fragmented and biotinylated. The labelled single-stranded DNA was hybridised for 16 hours at 45 °C on the Affymetrix HTA 2.0 microarrays following the manufacturer’s protocol. Subsequently, the microarrays were washed and stained with a streptavidin-phycoerythrin conjugate in an Affymetrix Fluidics Station 450. Signal amplification with antibodies was applied. The microarrays were scanned with a GeneChip Scanner 3000 7G (Affymetrix, CA, USA). Raw data were extracted from the scanned images using the Affymetrix GeneChip Command Console (AGCC) software version 4.0.

Preprocessing and quality control of the microarray data were done via the Expression Console 1.3.1 software (Affymetrix). For passing the quality control, signal intensities of hybridisation controls had to be within bounds. The robust multi-array average (RMA) algorithm^37^ was used with default parameters to perform background correction, quantile normalisation and log2 transformation of the raw fluorescence intensity values. Probe set summarisation was realised by the software according to two standardised workflows. For the “gene level analysis”, each probe set contains all probes matching a single gene (transcript cluster; TC). For the “exon level analysis”, each probe set matches a single exon fragment or splice junction. Each resulting expression profile consisted of 70,523 gene level probe sets and 914,585 exon level probe sets. The gene level profiles included 44,699 probe sets for coding transcripts, 22,829 probe sets for non-coding transcripts and 2,995 other probe sets (e.g., hybridisation controls). The raw and preprocessed HTA 2.0 microarray data are publicly available at NCBI’s Gene Expression Omnibus (GEO) database (accession numbers GSE73079 and GSE73080).

### Filtering of Differential Expressed Genes

The gene level profiles of the samples from the 10 patients at the 3 time points were analysed for differentially expressed genes during fingolimod treatment using the Affymetrix Transcriptome Analysis Console 1.0 (TAC) software. The filtering was done by comparing the gene expression levels immediately before the initiation of fingolimod therapy with those after 24 hours and after 3 months. Fold-changes (FC) and t-test p-values were calculated to determine significantly differentially expressed genes. A p-value threshold of 0.001 was used, which is more stringent than the default value of 0.05 in TAC. For FC, a cut-off at ±2.0 was chosen. The FC is the ratio of the gene expression at baseline and during therapy. It is calculated by TAC on the expression values in linear scale. Accordingly, a FC of −2.0 corresponds to RNA levels reduced by 50% and a positive FC of +2.0 corresponds to RNA levels increased by 100% in response to therapy.

### Analysis of Gene Functions

We examined to what extent genes that were found to be differentially expressed during therapy are involved in specific biological processes. For this purpose, we performed a gene set enrichment analysis using functional categories of the database Gene Ontology (GO). We used GOstats^38^, a R/Bioconducter software package, to calculate p-values and odds ratios (OR) for overrepresentation of GO gene sets in the list of differentially expressed genes. As a reference gene list, we used all measured genes that were expressed by CD4+ cells. Genes with an expression value <4 for all samples were not considered to be expressed, eliminating 26,268 of the 70,523 gene level probe sets from this analysis. To confine the result to specific gene groups, only GO terms of the categories “biological process” and “molecular function” with less than 200 members in the reference list (size) were tested. Moreover, GO terms comprising less than 4 differentially expressed genes (count) and GO terms with OR <7 were discarded.

### S1P Pathway Analysis

Another objective was the study of molecular interactions within the S1P-associated pathways. Therefore, we constructed a model of the S1P pathway, which we have described in more detail elsewhere^39^, to investigate the extent to which pathway components are modulated at the transcript level in response to therapy. Briefly, genes involved in S1P signalling were gathered from 11 review articles to compile a consensus pathway. This pathway includes the synthesis of natural S1P and the conversion of fingolimod as well as the interactions with S1P receptors. Moreover, the chemokine receptor CCR7, which plays a critical role for the homing of lymphocytes, was integrated. The S1P pathway was then visualised with the open source software Cytoscape 3.1.0^40^, where nodes (e.g., genes) and edges (e.g., interactions) are the building blocks of the molecular network. Changes in CD4+ cell expression after 3 months of fingolimod treatment compared to baseline were highlighted in colour in the pathway.

### Analysis of Alternative Splicing

Research has shown that the tens of thousands of human genes produce more than a hundred of thousands of different transcript isoforms. For instance, exons of a gene may be included within or excluded from the final mRNA (exon skipping). A major advantage of the high-resolution design of Affymetrix HTA 2.0 microarrays is that measuring and analysing transcript isoforms has been made possible. When analysing the data of the >6 million oligonucleotide probes with the exon level workflow, 914,585 probe sets (PSR and JUC) can be distinguished. This facilitates the examination of different transcriptional variants. If all the exons of a certain alternative splice variant have high signal intensities, we can infer that this transcript variant is expressed. As an example, we visualised the baseline data of patient Pat07 for the gene CX3CR1 at the exon level.

## Results

### Patients and Sample Information

Most RRMS patients showed a benefit from fingolimod treatment. Within a clinical follow-up period of one year, only two patients (Pat06 and Pat08) still had relapses. These two patients also showed a rapid disease progression in the first 12 months, whereas the remaining patients did not show a marked worsening of disability according to the EDSS (Table 1). Most patients continuously received fingolimod treatment during the one-year follow-up. Only Pat08 discontinued the therapy after 8 months and switched to alemtuzumab after 13 months.

The number of lymphocytes circulating in the blood of the patients decreased significantly after the start of fingolimod treatment. After 24 hours and 3 months, the numbers of separated CD4+ cells were reduced, on average, by >20% and >90%, respectively. The quality of cellular RNA was assessed by Bioanalyzer and estimated as RIN. The average RIN of all 30 samples obtained before and during fingolimod therapy was 9.3. Therefore, sufficient amounts of high quality RNA were available for the microarray analysis. Positive selection of CD4+ cells from whole blood was confirmed by high mRNA levels of CD4 in all samples (Supplementary Fig. S1).

### Transcriptome Dynamics

**Figure 1:**
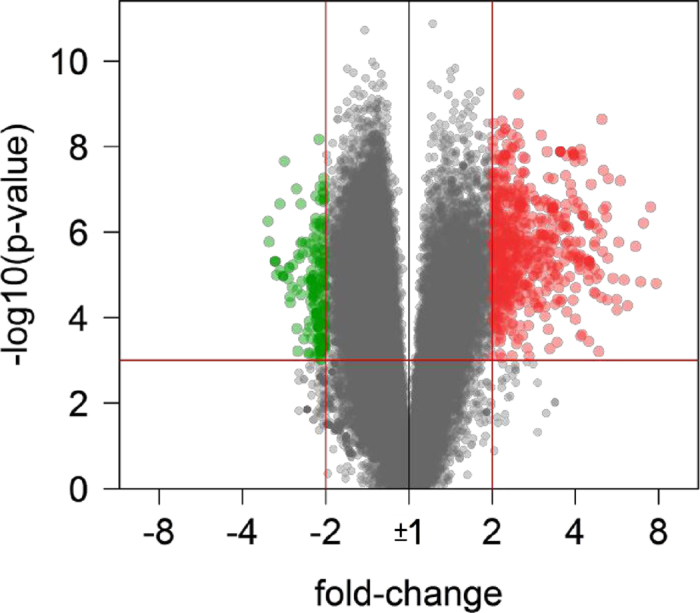
Volcano plot of gene expression changes. The transcriptome of circulating CD4+ cells from RRMS patients receiving fingolimod therapy for 3 months was compared to baseline. The x-axis specifies the fold-changes (FC) and the y-axis specifies the negative logarithm to the base 10 of the t-test p-values. Red vertical and horizontal lines reflect the filtering criteria (FC = ±2.0 and p-value = 0.001). FC > +2.0 indicates transcript levels increased by >100%, whereas FC < −2.0 indicates transcript levels reduced by >50%. Red and green dots represent probe sets for transcripts expressed at significantly higher (n = 618) or lower (n = 272) levels during therapy, respectively.

**Figure 2:**
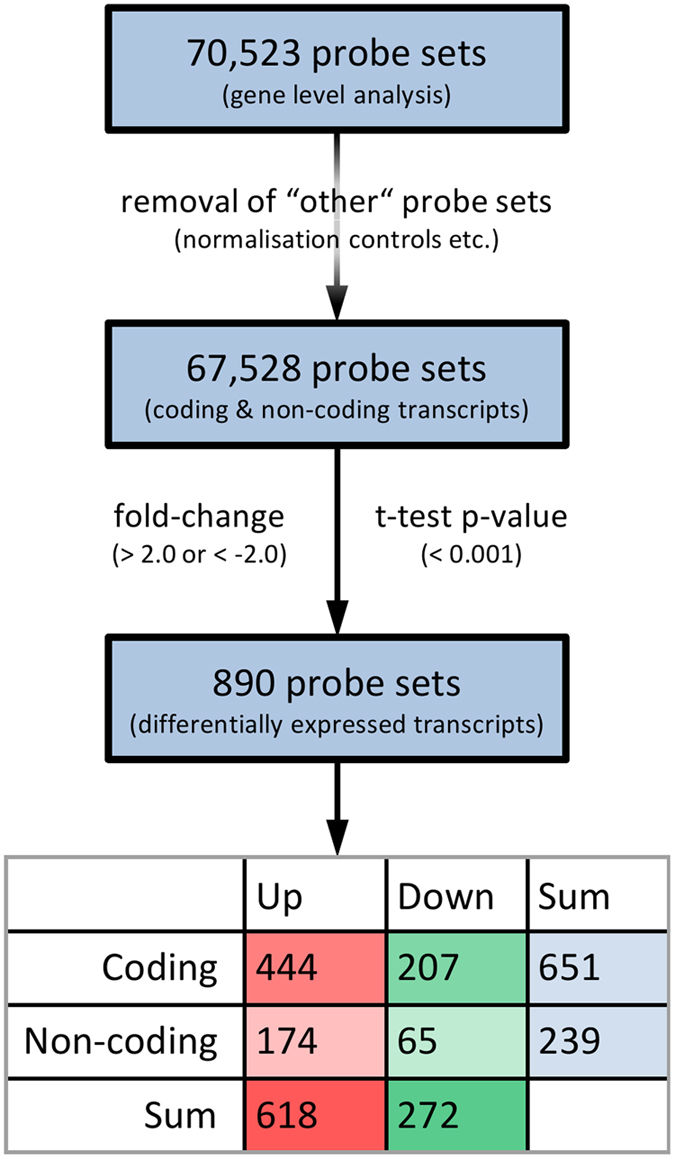
Identification of differentially expressed genes in CD4+ cells. In total, 890 probe sets were filtered when comparing the transcript levels after 3 months of therapy with the pre-treatment levels. This figure shows the number of all gene level probe sets (top), the number of probe sets after removal of normalisation controls (middle) and the number of probe sets for differentially expressed transcripts satisfying t-test p-value < 0.001 and fold-change > + 2.0 or <−2.0 (bottom). The table is giving the numbers of up-regulated (green) or down-regulated (red) and coding or non-coding transcripts.

Based on the data of the 70,523 gene level probe sets (transcript clusters), we searched for genes with significant expression changes in response to fingolimod therapy. None of the genes matched the filtering criteria (p-value < 0.001 and FC > + 2.0 or <−2.0) for the time point comparison of 24 hours after first administration of fingolimod versus baseline. In contrast, substantial transcriptome changes were seen after 3 months (Fig. 1). In total, 890 genes were filtered to be expressed at significantly higher levels (n = 618) or lower levels (n = 272) (Table 2, Supplementary Table S1). All of them had a false discovery rate <0.05 when correcting for multiple testing, and 651 genes were labelled as protein-coding and 239 genes were labelled as non-coding (Fig. 2).

**Figure 3:**
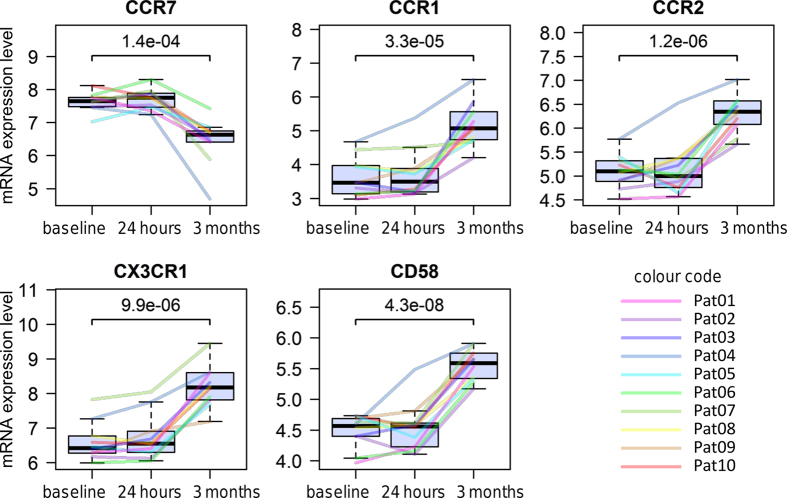
Longitudinal time course profiles of 5 selected genes. The figures show the individual gene expression levels at baseline as well as 24 hours and 3 months after fingolimod treatment initiation. Expression values are given in log2 scale as RMA normalised probe set summarised signal intensities. Different colours highlight the data for each of the 10 RRMS patients in the study. While CCR7 mRNA levels were reduced in CD4+ cells in response to fingolimod therapy, an elevated expression was measured for the other genes shown. The p-values were calculated by t-test.

The gene CCR7 (FC = −2.05), for example, was found to be expressed at lower levels and the genes CD58 (FC = 2.01), CX3CR1 (FC = 3.39), CCR1 (FC = 2.84) and CCR2 (FC = 2.36) were found to be expressed at higher levels in response to therapy in all of the 10 individual patients (Fig. 3). Moreover, several major histocompatibility complex (MHC) genes were significantly increased in expression after 3 months compared to pre-treatment levels, including HLA-DRA (FC = 4.70) and HLA-DRB1 (FC = 3.71). There were also 12 differentially expressed precursor miRNA (Table 3), including hsa-mir-216b (FC = −2.09), hsa-mir-142 (FC = 2.20) and hsa-mir-548c (FC = 2.73). Probe sets for these precursor miRNA contain oligonucleotide probes matching only to the sequence of the short stem-loop formed by the primary miRNA transcript. Interestingly, both the precursor miRNA hsa-mir-4668 (FC = 2.05) as well as the respective primary miRNA, the host gene UGCG (FC = 2.16), were identified to be expressed at increased levels during therapy.

### Associations to Gene Functional Categories

A GO term enrichment analysis was performed to functionally characterise the genes, which were identified to be differentially expressed in the course of therapy. For the two major GO categories “biological process” and “molecular function”, the top 10 GO terms associated to the filtered genes with OR >7 were determined. Overlapping and distinct gene sets were found to be significantly overrepresented (Table 4, Supplementary Table S1).

Twenty differentially expressed genes were assigned to the GO term “cytokine secretion” (GO:0050663, p-value = 1.50E-15, OR = 14.64). This GO term contains genes, which are related to processes that modulate the release of cytokines from cells. Of these 20 genes, CCR7 was lowered in expression, and the remaining 19 genes, including CSF1R, LYN, S100A12 and SYK, were elevated in expression during fingolimod treatment. A subset of 17 of these genes also belongs to the GO terms “protein secretion” (GO:0009306) and “regulation of cytokine secretion” (GO:0050707). The GO term “positive regulation of lymphocyte activation” (GO:0051251, p-value = 8.16E-13, OR = 7.18) contained 25 genes, e.g., LYN, PAK1 and TNFSF13B, which all showed enhanced expression in response to therapy.

The GO terms of the “molecular function” category “MHC class II receptor activity” (GO:0032395, p-value = 2.64E-11, OR = 89.81) and “IgG binding” (GO:0019864, p-value = 2.34E-08, OR = 66.86) were also highly enriched. From the list of differentially expressed genes, 8 human leukocyte antigen (HLA) class II genes and 6 Fc receptor genes (e.g., FCGR2A-C) were associated to these two gene sets. All of these genes were elevated in expression after 3 months of treatment relative to baseline. Similar and overlapping GO terms were identified to be significantly overrepresented (e.g., “IgG binding” and “immunoglobulin binding”) due to the hierarchical relationships in the GO database structure.

### Effects within the S1P Pathway

**Figure 4:**
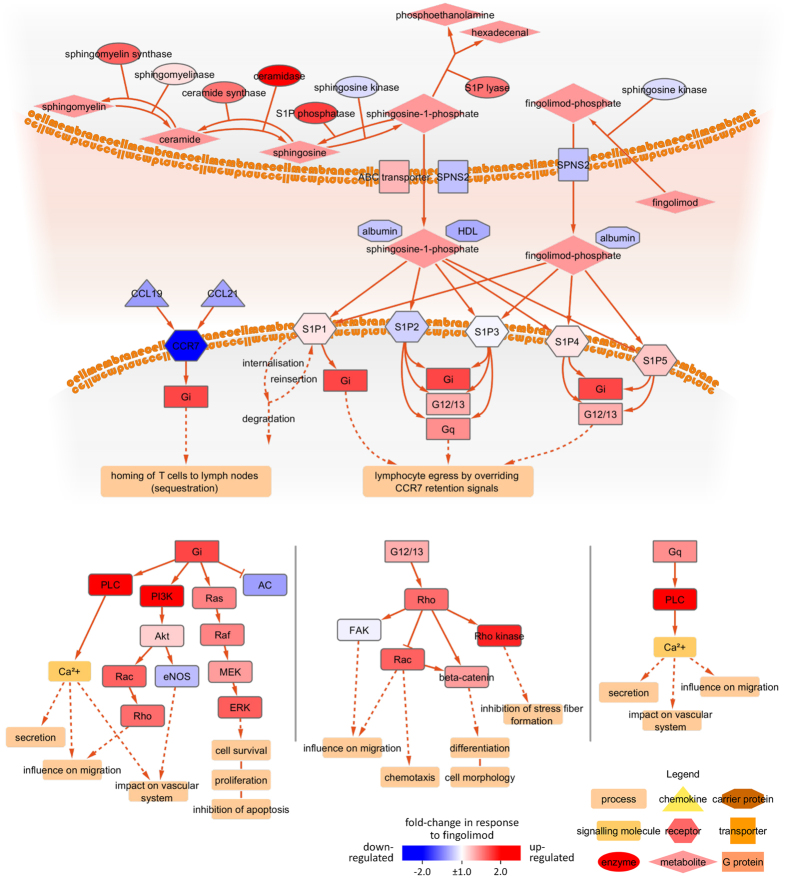
The molecular pathway modulated by fingolimod. Sphingosine-1-phosphate (S1P), a metabolite formed from sphingosine, is a major regulator of lymphocyte trafficking. As a structural analogue of sphingosine, fingolimod can be phosphorylated to fingolimod-phosphate, which binds to S1P receptors expressed by lymphocytes. However, while S1P leads to short-term internalisation of the S1P1 receptor, followed by reinsertion into the cell membrane, fingolimod-phosphate initiates a long-term internalisation, which leads to degradation of S1P1. This alters the chemotactic events in secondary lymphoid tissues. The S1P receptors usually associate with G proteins, which regulate a range of biological processes as presented in the three cascades in the lower part of this figure. Some pathway protein-coding genes were differentially expressed in CD4+ cells after 3 months of fingolimod treatment compared to baseline. The mRNA expression fold-change of each gene in the pathway is visualised in colour with up-regulated genes shown in red and down-regulated genes shown in blue. Different types of proteins (e.g., receptors and enzymes) are highlighted in different shapes.

Fingolimod is phosphorylated by sphingosine kinase 2 (SPHK2) to fingolimod-phosphate, which acts as an agonist on S1P receptors, especially S1P1. Binding of phosphorylated fingolimod to S1P1 results in internalisation of this receptor and loss of its signalling functions, leading to sequestration of lymphocytes in lymph nodes. The S1P-associated pathways are characterised by the interplay of numerous protein interactions and enzyme activities. The molecular network shown in Fig. 4 is a compilation of the physiological pathway for S1P synthesis as well as the therapeutic pathway with fingolimod^39^. Also shown is the competition between CCR7 and S1P receptor signalling, which have different chemotactic consequences. CCR7 engagement by its ligands, the chemokines CCL19 and CCL21, mediates the homing of naive T cells and TCM to lymph nodes, and the binding of S1P to S1P1 overrides this retention signal to promote lymphocyte egress. We analysed the changes in expression of the coding genes participating in the S1P pathway (Fig. 4). Certain components were expressed at higher levels (FC > + 2.0) three months after the start of fingolimod therapy (PI3K, PLC and ceramidase ASAH1). Rho kinases were notably elevated in expression as well (FC = 1.58 and FC = 1.87 for ROCK1 and ROCK2, respectively), whereas CCR7 was less abundant (FC = −2.05) relative to pre-treatment transcript levels. These changes reflect shifts in CD4+ cell subpopulations as well as regulatory effects, e.g., due to intracellular signalling and feedback mechanisms.

### Alternative Transcript Variants of CX3CR1

**Figure 5:**
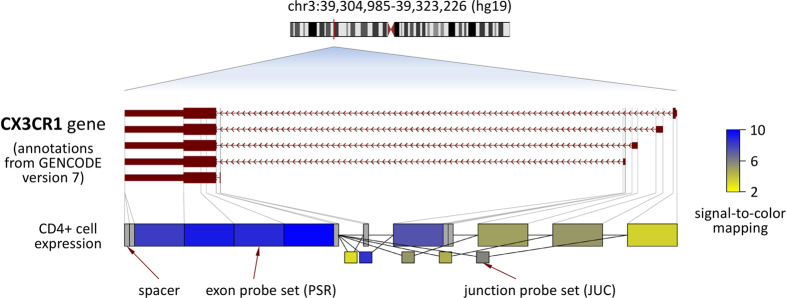
Exon level analysis for the CX3CR1 gene. In the upper part, the exon-intron structures of the five possible CX3CR1 mRNA variants generated by alternative promoter usage are visualised in red. Arrows in introns indicate transcription orientation. Below, the Affymetrix microarray data are visualised for CD4+ cells separated from a patient’s blood sample (Pat07) obtained at study onset. For each of the eight exon fragments there were ten 25mer oligonucleotide probes, and for each of the five probe sets for exon-exon splice junctions there were four probes. The processed probe set signals are in log2 scale, and blue colours indicate high expression while yellow colours indicate low expression. Spacers refer to sequences for which no corresponding probe exists. The longest CX3CR1 transcript, which results when the most distal 5′ transcription start site is used, is expressed at much lower levels than shorter variants.

To demonstrate the high coverage of the HTA 2.0 microarrays used in this study, we visualised the CD4+ cell gene expression data of an exemplary gene, CX3CR1, at exon level. This gene is encoded on chromosome 3. Five transcript variants of this gene are known to result from alternative transcription start sites. Figure 5 shows the signal intensities summarised from probes matching to distinct exon fragments and exon-exon splice junctions. The shorter transcript isoforms of CX3CR1 were found to be expressed at higher levels in comparison to the longest isoform.

## Discussion

To our knowledge, this is the first study investigating the transcriptome changes in response to fingolimod therapy in peripheral blood CD4+ cells of RRMS patients. For this purpose, we used high-resolution, high-coverage Affymetrix HTA 2.0 microarrays with >6 million probes for protein-coding and non-coding genes. Fingolimod administrated once daily reduces peripheral lymphocyte counts within a few hours after the first dose, but this initial effect is transient and normal values appear after one day^41^,^42^. Homing of lymphocytes to lymph nodes over longer periods is established only after continued dosing of fingolimod. Over a two-week period, the lymphocyte counts continue to decrease, reaching a nadir at 20–30% of baseline values^21^. This correlates with the transcriptome dynamics as changes in expression were seen in our study only after 3 months. No gene matched our filtering criteria after 24 hours, but after 3 months of treatment, we identified 890 genes to be significantly differentially expressed (Fig. 1, Supplementary Table S1). The majority of the genes (n = 618) were expressed at higher transcript levels during therapy.

As discussed in the following, we defined a comprehensive signature of therapy-responsive genes in circulating CD4+ cells and detected gene expression changes consistent with previous studies in the literature. For instance, as expected, the chemokine receptor CCR7 was significantly reduced in expression after 3 months of fingolimod therapy (FC = −2.05). CCR7 is responsible for controlling lymph node homing of naive T cells and TCM, including the Th17 population^29^,^43^. Fingolimod leads to the retention of these CCR7+ T cells in lymph nodes by prolonged internalisation of S1P receptors. CCR7- TEM, however, preferentially keep circulating in the peripheral blood^44^,^45^.

Many fingolimod-responsive genes identified in this study are immune-related genes, including also the chemokine receptors CX3CR1 (FC = 3.39) and CCR2 (FC = 2.36) (Figs 3 and 5). CX3CR1 is a transmembrane receptor involved in the recruitment of leukocytes to peripheral tissues and lymphoid organs^46^. Its binding chemokine CX3CL1 is implicated in the pathogenesis of several diseases such as rheumatoid arthritis^47^. Increased CX3CL1 levels in the cerebrospinal fluid and a higher percentage of CX3CR1-expressing CD4+ T cells in the blood were found in RRMS patients in comparison to healthy controls^48^. Peripheral CX3CR1+ CD4+ cells have been described to produce GZMA and PRF1^47^. CX3CR1 and GZMA (FC = 3.20) both contribute to the transendothelial migration of CD4+ TEM^49^,^50^. The relative increase of TEM in the blood during fingolimod therapy^28^,^30^ likely explains the higher mRNA levels of these two genes in our data. CCR2 is also known to be required for lymphocyte migration and monocyte chemotaxis during inflammatory conditions^51^. It is a surface marker of long-term CD4+ TEM prepared for mounting rapid recall responses^52^. Recent studies postulate CCR2 inhibition in MS as a therapeutic approach, but there is also evidence for protective effects of CCR2 during CNS inflammation^51^. Yopp *et al*. demonstrated that fingolimod impacts CCR2-driven migration by activating ABC transporters and ALOX5^53^. In our data, both CCR2 and ALOX5 (FC = 2.32) showed increased transcript levels during fingolimod therapy. Future studies may investigate the protein levels of these genes for confirmation.

We found that several toll-like receptor (TLR) genes (e.g., TLR1, TLR2 and TLR4) were up-regulated in expression in CD4+ cells after 3 months of fingolimod treatment. TLRs play a crucial role in the immune response to pathogen-associated and damage-associated molecular patterns, leading to the activation of specific transcription factors^54^,^55^. As reviewed by Jin *et al*., TLR agonists exert diverse direct effects on T cells^56^. TLR2 (FC = 4.17) has been discussed to modulate the suppressive activity of Treg^57^,^58^. TLR2 stimulation also promotes the proliferation and IL-17 production of Th17 cells^59^, which in turn have been linked to the pathogenesis of MS and other autoimmunity disorders^10^. Notably, loss of TLR4 (FC = 2.94) in CD4+ T cells was found to abrogate disease symptoms in the animal model of MS, mainly through blunted Th17 and Th1 responses^60^. The precise mechanisms governing T cell survival following TLR4 stimulation thus deserve further investigation.

Another gene that presented increased mRNA levels in blood CD4+ cells of fingolimod-treated patients was CD58 (LFA-3) (FC = 2.01), a cell adhesion molecule that binds to CD2 (LFA-2). This interaction provides a co-stimulatory signal for T cell activation^61^, which is controlled by epigenetic changes at the chromatin level^62^. Elevated expression of CD58 in T cells was also documented after one month of natalizumab treatment^63^. The expression of CD58 was found to be reduced in the cerebrospinal fluid of MS patients compared to controls^64^. Moreover, previous studies reported single nucleotide polymorphisms (SNPs) in the CD58 gene locus to be associated with the risk of MS^65^. These SNPs are suspected to affect the processing of the miRNA hsa-mir-548ac, which is located in the first intron of CD58^66^. The stem-loop of hsa-mir-548ac was not represented on the microarrays, but we detected elevated levels of hsa-mir-548c, another member of this miRNA family, after 3 months of therapy.

Altogether, when comparing the levels at baseline with the levels after 3 months of fingolimod treatment, 12 probe sets matching to miRNA stem-loops were filtered (Table 3). The precursor miRNA hsa-mir-4668 (FC = 2.05) and its host gene UGCG (FC = 2.16) were both increased in expression. DLEU2 (FC = 2.59), the host gene of hsa-mir-16–1, was also up-regulated. Prior studies showed hsa-mir-16-1 to be down-regulated in CD4+ cells of MS patients^67^ and to be up-regulated during IFN-beta therapy^68^. During GA treatment and in our study during fingolimod treatment, hsa-mir-142 was found to be differentially expressed^69^. These aforementioned miRNA are thus promising biomarkers of disease and therapy^16^. So far there has been no study performed in MS investigating explicitly the expression changes of mature miRNA in blood cells during fingolimod treatment. Our findings require further validation using real-time polymerase chain reaction analyses with higher sensitivity than oligonucleotide-based measurements.

The gene set enrichment analysis (Table 4) revealed genes involved in diverse biological processes such as “cytokine secretion” and “positive regulation of lymphocyte activation” to be significantly enriched in the list of genes differentially expressed in circulating CD4+ cells after 3 months of fingolimod therapy. These functional groups included LYN (FC = 5.16) and PAK1 (FC = 2.67). LYN encodes a Src tyrosine kinase, which is involved in regulating the activation of PI3K and MAPK signalling cascades^70^. The serine/threonine p21-activated kinase encoded by PAK1 links Rho-related GTPases to the MAPK pathway and influences cell adhesion, migration and proliferation^71^,^72^. PIK3CG (FC = 2.13), MAPK (ERK) and Rho kinases are downstream components in the S1P-associated signalling network (Fig. 4)^39^. Further studies are needed to investigate the precise implications of the molecular interactions of LYN and PAK1 in the context of immunomodulatory treatments for MS.

Most of the genes identified as differentially expressed in response to fingolimod treatment are evidently related to the peripheral shift in the frequencies of CD4+ cell subpopulations as a consequence of the reduced numbers of naive T cells and TCM. On closer inspection, we focused on typical markers for Th1 cells (CCR5 and CXCR3), Th17 cells (CCR6 and CD161) and Treg (CD25 and FOXP3)^73^,^74^,^75^. In our data set, we could not detect significant expression changes for any of those markers within the first 3 months of therapy. However, we found increased ENTPD1 (CD39) (FC = 2.42) transcript levels. This is consistent with a previous study demonstrating that CD4+ T cells from the blood of fingolimod-treated MS patients are enriched in CD39-expressing Treg^76^. It has been reported that CD39+ Treg have suppressive effects on Th17 cells^77^. Our transcriptome data did not show evidence of a preferential reduction in the number of peripheral blood Th17 cells within the CD4+ subset during therapy, which has been controversially discussed in the literature^29^,^43^. We did not further characterise the isolated CD4+ cells using flow cytometry, but it should be recalled that a subset of monocytes also slightly express CD4^78^. Therefore, not only CD3+ CD4+ T helper cells but also a variable proportion of CD4+ CD14+ monocytes was contained in the isolated cell population (Supplementary Fig. S1). Elevated levels of CD14 mRNA after 3 months of fingolimod therapy (Supplementary Table S1) are thus indicative of an increased frequency of monocytes and a lower level of enrichment of T cells, when only CCR7- TEM are spared in the circulation. This, in part, affected the microarray data of our study. Subsequent studies may combine flow cytometric sorting and subpopulation-specific (or single-cell) transcriptome analysis to better address this issue.

The identification of molecular biomarkers that allow prediction of disease progression may enable physicians to estimate the course of MS, to identify patients more likely to be responsive to a certain treatment and to distinguish risk patients requiring more frequent monitoring. With regard to our study cohort, it is conceivable that the two patients with ongoing disease activity during fingolimod therapy (Pat06 and Pat08, Table 1) may have an altered individual gene expression signature, but our data did not clearly indicate this. Two recent studies suggested that flow cytometric analysis of lymphocyte subpopulations in peripheral blood of MS patients might be useful for predicting clinical response to fingolimod^28^,^79^. However, larger patient cohorts and longer clinical follow-up periods are needed to define and establish prognostic biomarkers.

In conclusion, 890 genes were identified to be expressed at significantly higher or lower transcript levels in response to fingolimod therapy within the CD4+ immune cell population. The filtered genes comprise genes involved in functional pathways of T cells as well as less characterised genes whose role in the immunology and treatment of MS merits further investigation.

## Additional Information

**How to cite this article:** Friess, J. *et al*. Fingolimod alters the transcriptome profile of circulating CD4+ cells in multiple sclerosis. *Sci. Rep.* **7**, 42087; doi: 10.1038/srep42087 (2017).

**Publisher's note:** Springer Nature remains neutral with regard to jurisdictional claims in published maps and institutional affiliations.

## Supplementary Material

Supplementary Figure S1

Supplementary Table S1

## Figures and Tables

**Table 1:** Clinical and demographic data of the 10 patients with MS.

Patient	Gender	Age in years	Disease duration in years	Previous treatment	Treatment gap in months	EDSS at baseline	EDSS after 12 months	Relapses during 12 months prior to fingolimod	Relapses during 12-month follow-up
Pat01	female	43	20	IFN-beta-1a sc	38	4.0	3.0	1	0
Pat02	female	26	3	IFN-beta-1b sc	1	1.5	2.0	2	0
Pat03	female	29	5	glatiramer acetate	<1	2.5	1.5	3	0
Pat04	female	43	1	IFN-beta-1a im	1	4.0	3.5	2	0
Pat05	female	45	12	IFN-beta-1a im	2	3.0	3.0	3	0
Pat06	female	33	2	glatiramer acetate	<1	2.5	5.5	2	2
Pat07	female	46	9	IFN-beta-1b sc	<1	4.0	4.0	1	0
Pat08	male	33	4	IFN-beta-1a sc	<1	3.0	6.0	1	3
Pat09	male	46	15	IFN-beta-1b sc	<1	3.5	2.0	1	0
Pat10	male	37	9	glatiramer acetate	1	5.5	4.0	1	0
Median (range)		40 (26–46)	7 (1–20)		1 (0–38)	3.3 (1.5–5.5)	3.3 (1.5–6.0)	1.5 (1–3)	0 (0–3)

The table provides gender, age at study onset, disease duration and previous treatment for each patient selected for the transcriptome profiling. The time span between the last injection of the previous immunomodulatory treatment and the first oral administration of fingolimod is given as well (treatment gap). Additionally, the EDSS and the number of relapses before the start of fingolimod therapy (baseline) as well as after a clinical follow-up period of one year are given. EDSS: Expanded Disability Status Scale, IFN: interferon, im: intramuscular, sc: subcutaneous.

**Table 2:** Top genes expressed at higher mRNA levels in response to fingolimod.

Probe set	Symbol	Entrez	Location	Probes	P-value	Fold-change
TC07001546.hg.1	FGL2	10875	chr7 (q11.23)	30	1.47E-05	6.896
TC11003451.hg.1	MS4A7	58475	chr11 (q12.2)	145	2.20E-06	6.610
TC0X000171.hg.1	CYBB	1536	chrX (p11.4)	168	5.26E-05	6.183
TC12003207.hg.1	CLEC12A	160364	chr12 (p13.2)	137	1.34E-05	6.016
TC12000591.hg.1	IRAK3	11213	chr12 (q14.3)	183	1.72E-06	5.731
TC12001796.hg.1	DUSP6	1848	chr12 (q21.33)	139	6.67E-05	5.665
TC01001346.hg.1	MNDA	4332	chr1 (q23.1)	103	3.77E-05	5.650
TC12001215.hg.1	CLEC7A	64581	chr12 (p13.2)	208	1.21E-05	5.474
TC02000398.hg.1	PLEK	5341	chr2 (p13.3)	140	5.60E-08	5.268
TC12000309.hg.1	LRRK2	120892	chr12 (q12)	649	4.08E-06	5.240
TC11001839.hg.1	MS4A6A	64231	chr11 (q12.2)	233	4.46E-07	5.176
TC08000383.hg.1	LYN	4067	chr8 (q12.1)	240	2.54E-07	5.160
TC17000103.hg.1	CD68	968	chr17 (p13.1)	110	2.98E-07	5.079

Gene expression in peripheral blood CD4+ cells was analysed in RRMS patients receiving fingolimod therapy. After 3 months of treatment, 890 genes were identified to be differentially expressed relative to pre-treatment levels (Supplementary Table S1). A subset of 13 probe sets representing protein-coding genes was filtered even with fold-change > + 5.0. This table gives the Affymetrix HTA 2.0 transcript cluster (TC) probe set identifier, the official gene symbol, the Entrez database identifier, the genomic location, the number of 25mer oligonucleotide probes for each probe set as well as t-test p-values and fold-changes.

**Table 3:** Differentially expressed precursor microRNA in the CD4+ cell population.

Probe set	Symbol	Entrez	Location	Probes	P-value	Fold-change
TC17000728.hg.1	hsa-mir-21	406991	chr17 (q23.1)	30	4.18E-07	4.376
TC17001729.hg.1	hsa-mir-142	406934	chr17 (q22)	30	1.18E-05	2.204
TC02001872.hg.1	hsa-mir-216b	100126319	chr2 (p16.1)	30	8.72E-08	−2.093
TC12000573.hg.1	hsa-mir-548c	693129	chr12 (q14.2)	30	7.24E-05	2.731
TC07000452.hg.1	hsa-mir-590	693175	chr7 (q11.23)	30	4.21E-05	2.641
TC10000348.hg.1	hsa-mir-605	693190	chr10 (q21.1)	30	8.41E-07	−2.058
TC20000241.hg.1	hsa-mir-644a	693229	chr20 (q11.22)	30	7.74E-08	3.846
TC04001648.hg.1	hsa-mir-3140	100422896	chr4 (q31.3)	30	2.25E-05	2.083
TC10000815.hg.1	hsa-mir-4295	100422909	chr10 (q25.2)	30	1.08E-05	2.037
TC02000369.hg.1	hsa-mir-4434	100616419	chr2 (p14)	10	1.24E-04	2.259
TC05000673.hg.1	hsa-mir-4461	100616209	chr5 (q31.1)	30	2.13E-04	−2.055
TC09000561.hg.1	hsa-mir-4668	100616114	chr9 (q31.3)	25	1.27E-05	2.051

This table shows 12 precursor miRNA that were identified as more abundant (n = 9) or less abundant (n = 3) after 3 months of fingolimod treatment in comparison to baseline (p-value < 0.001 and fold-change > + 2.0 or <−2.0). For each miRNA stem-loop, the table provides the miRBase database symbol, the Entrez database identifier, the genomic location, the Affymetrix HTA 2.0 probe set and the number of probes on the microarrays that were used to quantify the expression.

**Table 4:** Gene set enrichment analysis of differentially expressed genes.

GO accession	GO term description	Count	Size	P-value	OR
Biological processes					
GO:0002429	immune response-activating cell surface receptor signaling pathway	28	183	6.14E-16	8.68
GO:0050663	cytokine secretion	20	84	1.50E-15	14.64
GO:0050707	regulation of cytokine secretion	17	70	1.49E-13	14.87
GO:0009306	protein secretion	23	152	3.49E-13	8.41
GO:0051251	positive regulation of lymphocyte activation	25	190	8.16E-13	7.18
GO:0060333	interferon-gamma-mediated signaling pathway	16	72	3.48E-12	13.19
GO:0071346	cellular response to interferon-gamma	17	89	9.80E-12	10.92
GO:0019886	antigen processing and presentation of exogenous peptide antigen via MHC class II	16	79	1.58E-11	11.71
GO:0034341	response to interferon-gamma	18	105	1.61E-11	9.60
GO:0030595	leukocyte chemotaxis	18	106	1.91E-11	9.49
Molecular functions					
GO:0032395	MHC class II receptor activity	8	12	2.64E-11	89.81
GO:0019864	IgG binding	6	10	2.34E-08	66.86
GO:0019865	immunoglobulin binding	7	17	4.23E-08	31.30
GO:0004896	cytokine receptor activity	11	65	1.80E-07	9.21
GO:0004982	N-formyl peptide receptor activity	5	9	6.39E-07	55.51
GO:0050664	oxidoreductase activity, acting on NAD(P)H, oxygen as acceptor	5	13	6.06E-06	27.74
GO:0008329	signaling pattern recognition receptor activity	5	14	9.25E-06	24.66
GO:0038187	pattern recognition receptor activity	5	14	9.25E-06	24.66
GO:0003823	antigen binding	7	47	7.69E-05	7.81
GO:0019955	cytokine binding	7	51	1.31E-04	7.09

Listed are functional groups associated to the genes differentially expressed in peripheral blood CD4+ cells of MS patients during fingolimod treatment. The analysis was done using the GOstats R/Bioconductor software package and Gene Ontology (GO) terms of the categories “biological process” and “molecular function”. Only the top 10 GO terms according to the p-value with odds ratio >7, count >4 and size <200 are shown per category. For instance, the GO term “cytokine secretion” (GO:0050663) was found to be overrepresented. In total, 84 expressed genes belonged to this term (size). Of those, more genes than expected by chance (count = 20) corresponded to the 890 filtered probe sets. According to odds ratio (OR = 14.64) and p-value (1.50E-15), “cytokine secretion” genes are thus enriched in the list of genes, which are expressed at significantly higher or lower transcript levels after 3 months of fingolimod therapy.
