# Supplementary material for: Fingolimod alters the transcriptome profile of circulating CD4+ cells in multiple sclerosis
Source: Sci Rep. 2017 Feb 3;7:42087. doi: 10.1038/srep42087 (PMC5290459; doi:10.1038/srep42087)
Supplement: Supplementary Figure S1 [file srep42087-s1.pdf]

Supplementary Figure S1

Journal: Scientific Reports
Title: Fingolimod alters the transcriptome profile of circulating CD4+ cells in multiple sclerosis
Authors: Friess J, Hecker M, Roch L, Koczan D, Fitzner B, Angerer IC, Schröder I, Flechtner K, Thiesen H-J, Winkelmann A, Zettl UK
Correspondence: Michael Hecker, University of Rostock, Department of Neurology, Rostock, Germany; michael.hecker@rocketmail.com

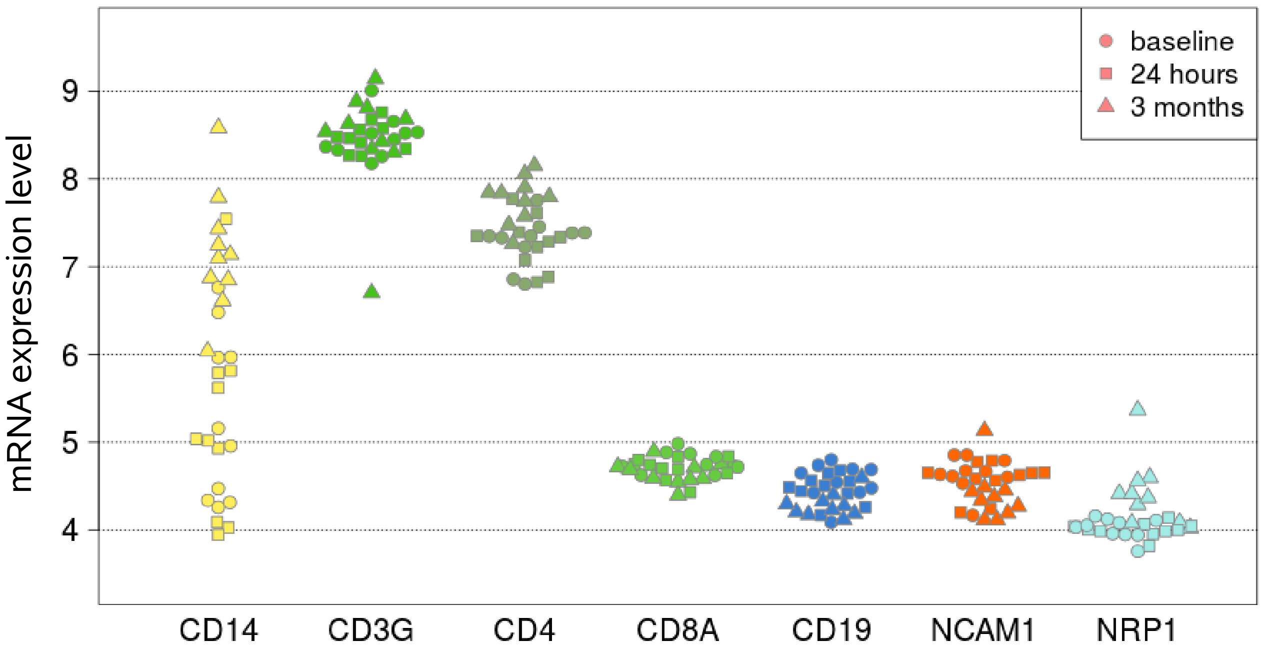

Analysis of the purity of the isolated CD4+ cells. We visualized the measured transcript levels of seven selected genes, which are relatively specifically expressed in different blood cell types, namely CD14 (monocytes), CD3G (T cells), CD4 (T helper cells), CD8A (cytotoxic T cells), CD19 (B cells), NCAM1 (natural killer cells) and NRP1 (dendritic cells). The processed probe set signals for each gene are in log2 scale. CD4 was expressed at high levels (range, 6.80-8.15) in all 30 samples of the microarray data set, similar to CD3G (range, 6.70-9.14), whereas the other genes were usually expressed at low levels (<5.00). The elevated expression of CD14, however, indicates the presence of CD4+ monocytes. This demonstrates that CD4+ cells were successfully positively selected, and that this cell population contains CD3+ T cells as well as a variable fraction of CD14+ monocytes.
